# Predictive Modeling of Sequential Fermentation for Sustainable Date Vinegar Production From Unripe Kharak Dates

**DOI:** 10.1002/fsn3.71605

**Published:** 2026-04-10

**Authors:** Fatemeh Rasi, Morteza Khomeiri, Seid Mahdi Jafari, Mahdi Kashaninejad, Alireza Sadeghi

**Affiliations:** ^1^ Faculty of Food Science and Technology Gorgan University of Agricultural Sciences and Natural Resources Gorgan Iran; ^2^ Halal Research Center of IRI, Iran Food and Drug Administration Ministry of Health and Medical Education Tehran Iran

**Keywords:** acetic starter, *Acetobacter*, agricultural waste, fermentation, fermentation kinetics

## Abstract

This study optimizes sequential fermentation and predictive modeling for sustainable, high‐quality vinegar production from unripe Kharak dates, an underutilized byproduct. Acetic acid bacteria (AAB) were isolated from Tehran and Gorgan samples (Iran), identified as *Acetobacter pasteurianus* , *A. aceti* , and *A. tropicalis* via morphological, biochemical, and PCR methods. Alcoholic fermentation was done by *Saccharomyces cerevisiae* at 20%, 30%, and 40% date syrup concentrations, followed by acetic fermentation with AAB. Optimal yeast growth and alcohol yield occurred at 20%, while *A. pasteurianus* showed robust performance across all levels, with 30% date syrup concentration yielding the best balance of growth, acidity (2.3% w/v), and pH stability. Gaussian, Gompertz, Rational, Sinusoidal Fit, and Logistic models effectively described microbial dynamics. This work enhances byproduct valorization and supports scalable, dietary‐friendly vinegar production.

## Graphical abstract

Predominant acetic acid bacteria from date vinegar were successfully isolated and identified. Introduced and optimized Kharak vinegar production using isolated acetic acid bacteria. Identified *A. pasteurianus* as the most effective isolate for vinegar production.

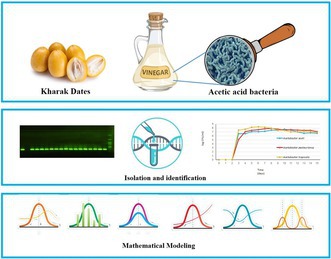

## 1 Introduction

Date vinegar (DV), produced from date syrup especially unripe Kharak dates, is a traditional Middle Eastern condiment valued for its unique flavor, nutritional profile, and potential health benefits (Ali et al. 2022; Nosratabadi et al. 2024). The global vinegar market reached US$2.27 billion in 2021 and is projected to grow by 2.6% annually through 2027 (Kourouma et al. 2023). DV, produced from date syrup, particularly from unripe Kharak dates, offers a sustainable use of agricultural byproducts, enhancing both cultural and economic value (Nosratabadi et al. 2024). DV offers a range of potential health benefits derived from its phenolic compounds, organic acids, and fermentation by‐products. Specifically, it may support glycemic control by reducing glycated hemoglobin (HbA1c) and fasting blood sugar (FBS), improve lipid profiles by lowering total cholesterol (TC), low‐density lipoprotein (LDL), and triglycerides (TG) while increasing high‐density lipoprotein (HDL), reduce inflammation by decreasing C‐reactive protein (CRP) and tumor necrosis factor alpha (TNFα), and exhibit antioxidant and antimicrobial effects (Al‐Qaisi et al. 2024; Ali et al. 2022, 2019, 2019; Hegazy et al. 2024; Tang, Zhu, et al. 2024).

The production process involves clarification and stabilization to ensure quality and safety, yielding a transparent red‐black vinegar with a specific gravity of 1.18, total acidity of 4.98% (w/v), minimal alcohol content, and 0.17% ash (Perumpuli and Dilrukshi 2022). The fermentation of DV is a complex process involving yeast‐mediated conversion of sugars to ethanol, followed by oxidation to acetic acid by acetic acid bacteria (AAB). This process is influenced by parameters such as temperature, time, and starter culture concentration (S. Li et al. 2015). Fermentation technology, one of the oldest food preservation methods, enhances the nutraceutical value, flavor, and functionality of foods like bread, yogurt, and alcoholic beverages (Choudhary et al. 2018). Recent advances in fermentation technology and modeling have provided opportunities to optimize this process, improving efficiency and product quality.

Kinetic models, such as Logistic, Gompertz, Richards, von Bertalanffy, and DoseResp, are critical for understanding microbial growth dynamics and optimizing fermentation conditions (Wang, Song, et al. 2022). These models can show how a particular microbial community, which may be composed of a few or several dominant taxonomic groups, interacts within a complex network architecture. Optimization and modeling of vinegar fermentation conditions have been extensively studied (Chakraborty et al. 2018; Ghosh et al. 2012; Nyuykongi et al. 2023; Román‐Camacho et al. 2023; Selvanathan and Masngut 2023; Song et al. 2022; Tang, Wang, et al. 2024; Tesfaye et al. 2002; Yang et al. 2024). However, specific factors affecting DV fermentation, particularly with unripe Kharak dates, remain underexplored. This study aims to model the fermentation process within optimal temperature, time, and starter inoculation ranges, using Kharak dates to produce a sustainable, high‐quality vinegar.

## 2 Materials and Methods

### 2.1 Materials

Unripe dates (*Phoenix dactylifera*, Kharak variety) were collected from the groves of Shahrebabak (Kerman, Iran) during the harvesting season (July–August) and stored at −20°C for up to 3 months prior to use (viability confirmed by post‐thaw microbial counts > 95%). Commercial kits were: GeneJet Genomic DNA Purification Kit (analytical grade, Thermo Fisher Scientific, USA), AccuPrep PCR Purification Kit (analytical grade, Bioneer, South Korea), and DNA Safe Stain (analytical grade, Sinaclon, Iran). All other chemicals used in this study were of analytical grade and purchased from chemical suppliers.

### 2.2 Analysis of Date Compounds

Date proximate analysis was conducted in triplicate following AOAC standards to ensure robustness, including moisture (AOAC‐976.06), ash (AOAC‐915), protein (AOAC 990.03), and fat content (AOAC 954.02). Samples were homogenized using a laboratory blender (Model XYZ, Waring, USA) at 10,000 rpm for 2 min to ensure uniformity prior to analysis. Fiber content was determined using the enzymatic‐gravimetric method (AOAC 59), while iron content was analyzed by atomic absorption spectrophotometry (AOAC‐999.11).

### 2.3 Alcohol Production Using Yeast

**FIGURE 1:**
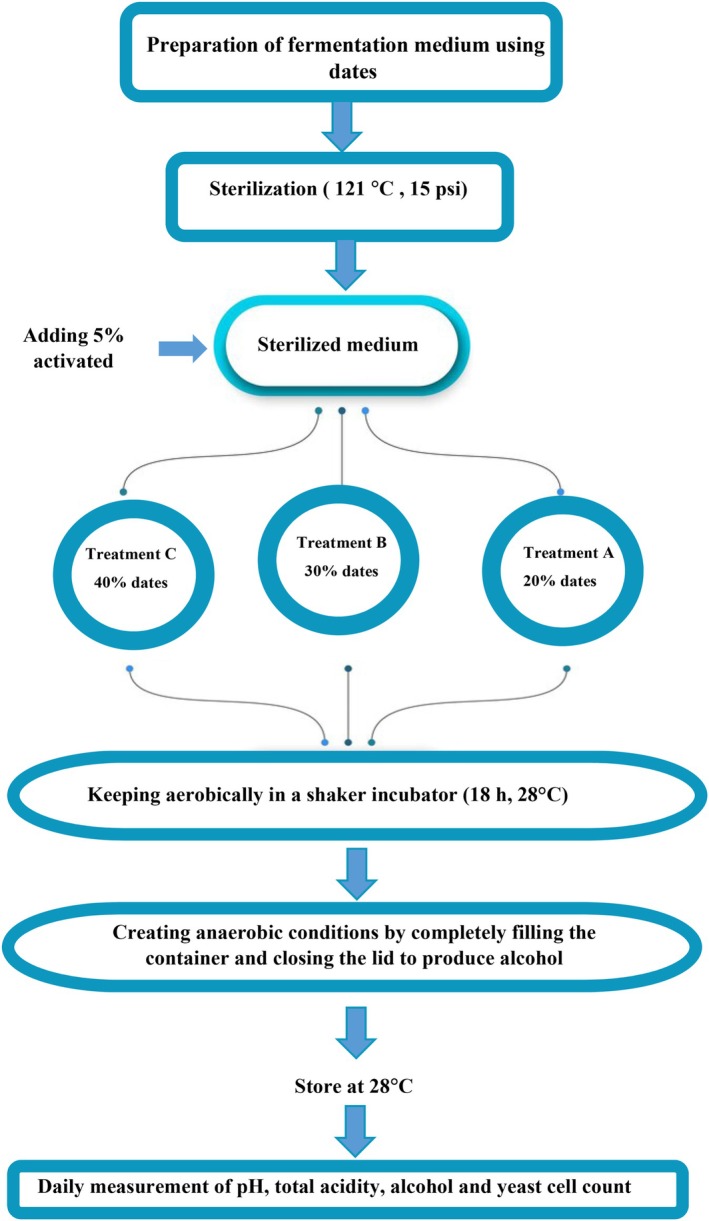
Changes in yeast cell count, alcohol content, acidity (as AA), and pH during the alcoholic fermentation stage of date vinegar production.

*Saccharomyces cerevisiae* (PTCC 5052) from the Persian Type Culture Collection of Iran was used for alcoholic fermentation. The yeast was activated in Yeast Malt (YM) medium, comprising 10 g glucose, 5 g soy peptone, 3 g yeast extract, and 3 g malt extract/L distilled water, with pH adjusted to 6.2 ± 0.2. After 24 h activation at 28°C, the yeast was washed and prepared as a microbial suspension (10^8^ CFU/mL) (5% v/v inoculum, equivalent to 5 × 10^7^ CFU/mL initial load; all fermentations in triplicate). The microbial count was determined using the surface plate method on YM agar. The dates were crushed and added to water in 20%, 30%, and 40% by weight to facilitate fermentation. Fermentation was conducted in 2 L volumes under aerobic conditions at 28°C for 15 days (no stirring or shaking to mimic traditional processes). The pH was monitored daily using a calibrated pH meter but was not adjusted during fermentation. The suspension was inoculated into treatments (Table 1), with fermentation monitored for 15 days. Controls without yeast and dates were also evaluated for comparison (Figure 1).

**TABLE 1:** Treatments for adding yeast and bacterial suspensions.

First stage (alcoholic fermentation)	Second stage (acetic fermentation)
A: 20% date + yeast	A1: 20% date + isolate 1
	A2: 20% date + isolate 2
	A3: 20% date + isolate 3
B: 30% date + yeast	B4: 30% date + isolate 1
	B5: 30% date + isolate 2
	B6: 30% date + isolate 3
C: 40% date + yeast	C7: 40% date + isolate 1
	C8: 40% date + isolate 2
	C9: 40% date + isolate 3

### 2.4 Cultivation of Acetic Acid Bacteria

Thirty AAB isolates were cultured in GYC medium (100 g/L glucose, 10 g/L yeast extract, 20 g/L calcium carbonate, 15 g/L agar, initial pH 6.8 ± 0.1) at 30°C for 7 days under static aerobic conditions. Growth rates were monitored by measuring the optical density (OD) at 600 nm every 24 h, with microbial counts determined using the surface plate method on GYC agar in triplicate. Acidity, pH, and alcohol consumption were evaluated daily in triplicate throughout the incubation period. Among them, 12 isolates exhibiting the highest growth rates, efficient alcohol consumption (≥ 80% reduction), significant acid production (≥ 2.0% w/v acidity), and lower pH (drop ≥ 1.0 unit) were selected for further comparison.

### 2.5 Preparation of Vinegar From Alcoholic Solution

**FIGURE 2:**
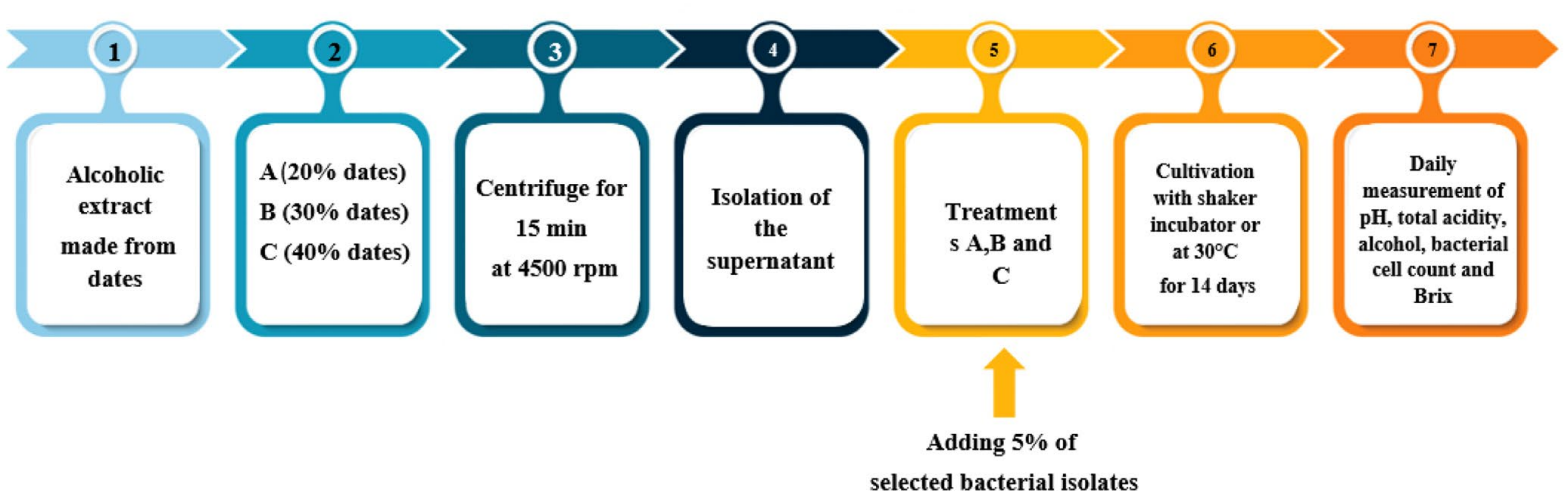
Changes in bacterial cell count, alcohol content, acidity (as AA), total soluble solids (TSS), and pH during the acetification stage of date vinegar production.

Thirty AAB isolates were screened for vinegar production by inoculating them into the alcoholic solution after completion of alcoholic fermentation. The bacterial suspension (5% v/v inoculum, 10^8^ CFU/mL; *n* = 3, determined via the surface plate method on GYC agar) was added to 1 L alcoholic solution, followed by incubation under aerobic conditions at 30°C for 15 days (no stirring to promote surface acetification by AAB). The pH was monitored daily using a calibrated pH meter but was not adjusted during fermentation. Bacterial growth, alcohol consumption, acidity, and pH were monitored daily throughout this period, all measurements in triplicate. These isolates were grown in tubes containing GY (Glucose‐Yeast extract) broth, incubated at 30°C for 5–7 days, and adjusted to McFarland 0.5 concentration (B. Wang et al. 2024). The selected AAB isolates were activated in a medium containing 5 g glucose, 2.5 g malt extract, and 1 g peptone/L distilled water, supplemented post‐autoclaving with 4.5% AA and 3.5% ethanol. The bacteria were incubated at 30°C for 2–3 days. A microbial suspension with 10^8^ CFU/mL was prepared, and 5% of it was inoculated into the fermented alcoholic solution after the alcoholic fermentation phase was completed (Budiarti et al. 2022). Over the next 15 days, parameters such as bacterial cell count, alcohol content, acidity (as AA), TSS, and pH were measured daily, in triplicate (Figure 2). The AAB were added only after the alcoholic fermentation phase was completed, marking the start of the acetic fermentation phase.

### 2.6 Vinegar Analysis

Yeast and AAB cell counts were performed in triplicate using the surface plate method on YM and GYC agar media, respectively, to ensure robust enumeration. (Wang, Rutherfurd‐Markwick, et al. 2022). Triplicate plating was conducted for accuracy. The pH of samples was determined using a pH meter, and acidity was measured according to AOAC 745–744 using titration with 0.1 N NaOH to phenolphthalein endpoint in triplicate.

TSS was measured using a desktop refractometer (Model XYZ, ATAGO Co. Ltd., Japan), calibrated at room temperature with standard sucrose solutions (0%–50% w/v) in triplicate. Alcohol content was assessed using the potassium dichromate titration method, where ethanol was oxidized to AA, and the endpoint was determined by the disappearance of the blue color in a starch‐iodine indicator (Joshi et al. 2019) in triplicate, verified against a standard curve prepared with known ethanol concentrations (0%–10% v/v). Ethanol and acetic acid concentrations were tracked via these titration methods; no gas chromatography was used. Alcohol consumption was calculated as the reduction in alcohol content from initial levels.

### 2.7 Isolation and Identification of Acetic Acid Bacteria

In order to culture AAB, the surface culture method was used. In this method, from the prepared dilutions, two repetitions were cultured (0.1 mL on each plate) on the GYC culture medium (10% glucose, 1% yeast extract, 2% calcium carbonate, 1.5% agar, pH = 6.8). Then it was incubated at 30°C for 1 week (Gerard et al. 2020). Thirty colonies were isolated based on their morphological characteristics, including color, shape, and colony elevation, from diluted samples (10^−3^ to 10^−4^). The selected colonies were cultured in GYC medium and stored for long‐term maintenance in glycerol stocks at −80°C. A total of 30 colonies were purified by GYC medium and kept in two short‐term and long‐term methods (De Vero et al. 2006). For short‐term storage, GYC culture medium was prepared by slope culture as a stock culture, incubated for 2–5 days at 30°C, then transferred to the refrigerator and recultured every 4 weeks. For long‐term storage, 500 μL active culture of each bacterial isolate in GY medium (5% glucose, 1% yeast extract), along with 500 μL sterile 40% v/v glycerol solution, were added to Eppendorf tubes and immediately frozen in liquid nitrogen (Wang et al. 2024).

### 2.8 Verification Tests for Acetic Acid Bacteria

A drop of 3% H_2_O_2_ was added to fresh microbial spreads, and the formation of air bubbles was observed for 5 min. This catalase test was used to identify AAB by detecting the presence of the enzyme catalase, which breaks down H_2_O_2_ into water and oxygen, producing observable bubbles. This reaction distinguishes AAB, which are catalase‐positive, from other microorganisms (Adedayo and Mohammed 2024). Positive control used *Acetobacter aceti* (ATCC 15973), and negative control used *Lactobacillus plantarum* (ATCC 14917). Microbial spreads were stained with crystal violet, Lugol's solution, and safranin, and cell arrangement and Gram reaction were determined by a light microscope at 100× magnification by two independent observers to confirm results (Ramadhani et al.  2020). For long‐term storage, glycerol stocks were maintained at −80°C for up to 12 months, with viability assessed by culturing on GYC agar after thawing.

### 2.9 Antimicrobial Activity Evaluation (Inhibition Zone (IZ) Measurement)

Antimicrobial activity of selected isolates was evaluated using the agar diffusion method. Tests were conducted in triplicate on Mueller‐Hinton agar, with ampicillin (10 μg/disc) as the positive control and sterile water as the negative control. Sterile 6 mm filter paper discs were impregnated with bacterial cultures adjusted to 10^8^ CFU/mL (confirmed via surface plate method on GYC agar). Discs were placed on agar plates seeded with common pathogens (e.g., *E. coli* or *S. aureus* ). The inhibition zones (IZs) were measured after 24 h incubation at 30°C. The diameter of the zone, where no microbial growth occurred around the discs, was recorded as IZ, providing a quantitative measure of antimicrobial activity.

### 2.10 PCR and Amplification of rDNA 16 s Region

DNA extraction was carried out using the Fermentas kit (USA), with DNA concentration measured by NanoDrop spectrophotometry (Gerard et al. 2020). The 16S rDNA region was amplified in a final volume of 20 μL, containing the following optimized components: 10 μL PCR master mix (Bioneer, South Korea), 1 μL forward primer (5′‐AGAGTTTGATCMTGGCTCAG‐3′), 1 μL reverse primer (5′‐TACGGYTACCTTGTTACGACTT‐3′), 2 μL DNA template, and 6 μL nuclease‐free water. Each component of the reaction mixture was added to the microtubes, which were then placed inside the thermocycler and subjected to the temperature program outlined in Table 2. To observe the results, the PCR amplicons were loaded into a 0.8% agarose gel and electrophoresed at 80 V for 35 min (Soumahoro et al. 2020). The expected PCR product length was approximately 1500 bp. PCR products were purified using the AccuPrep PCR Purification Kit (Bioneer, South Korea) before sequencing. PCR products were sequenced by Bioneer Co. (South Korea). Sequences were edited and aligned using BioEdit software and compared with the NCBI database using BLAST, with ≥ 97% similarity confirming species identification (Kubizniaková et al. 2021). To ensure the accuracy of the PCR amplification, both positive and negative controls were included in the experiment. The positive control consisted of a known DNA sample with a target sequence to confirm the effectiveness of the primers and the PCR conditions. The negative control contained no DNA template, ensuring that no contamination occurred during the PCR process. For size estimation of the PCR products, a DNA ladder (e.g., 100 bp DNA ladder, Thermo Scientific) was used, which spans from 100 bp to 1500 bp. The PCR amplicons were compared against this ladder to determine the size of the amplified fragments.

**TABLE 2:** The steps of performing the PCR.

Stages		Temperature (°C)	Time (min)	Cycle number
Initial denaturation		95	5	1
Temperature cycle	Denaturation	95	45	30
	Annealing	52	45	
	Extension	72	1	
Final extension		72	7	1

#### 2.10.1 Isolation and Characterization of Microbial Isolates for PCR Analysis

The microbial isolates were obtained from traditional vinegar sources and screened based on acidity and pH to select strains suitable for further analysis. Each isolate was labeled with a unique isolate code for tracking and PCR amplification. The acidity was determined by titrating the samples with 0.1 N NaOH to an endpoint, expressed as a percentage of AA. The pH of each isolate culture was measured using a calibrated pH meter to confirm environmental adaptation. Isolates were selected for PCR if they exhibited acidity ≥ 0.5% and a pH drop ≥ 0.5 units, indicating suitability for acetic fermentation. These values helped to identify and select isolates with distinct acidity and pH characteristics for subsequent PCR analysis and genetic identification.

### 2.11 Modeling

The growth of yeast and AAB during DV fermentation was modeled using Curve Expert 1.3 software. Variables such as date concentration (20%, 30%, 40%), temperature, pH, and time were included, and microbial growth rates, alcohol content, acidity, pH, and TSS (Brix) were measured over the 13‐day period. Inputs to the models comprised microbial growth data and biochemical parameters—specifically, alcohol content, acidity, pH, and TSS (Brix), which were recorded at regular intervals to accurately capture the dynamics of fermentation. Models included Gaussian, Gompertz, Rational, Sinusoidal Fit, and Logistic, evaluated using goodness‐of‐fit metrics R^2^ and standard error (S).

### 2.12 Statistical Analyses

Statistical analyses were performed by one‐way ANOVA. Assumptions of normality and homogeneity of variance were checked prior to ANOVA using the Shapiro–Wilk test and Levene's test, respectively. All experiments were done in triplicate; data reported as mean ± SD, and significance was determined at *p* < 0.05 using Duncan's multi‐range test. SPSS 16 software was used for statistical analysis, Excel 2010 for graphs, and Curve Expert 1.3 for modeling fermentation parameters.

## 3 Results and Discussion

### 3.1 Compositions of Dates

The biochemical composition of dates varies notably across different cultivars and stages of ripening. Dates are primarily composed of sugars, which constitute approximately 70%–80% of their dry weight. The main sugars found in dates are sucrose, glucose, and fructose. The specific composition of dates used in this study is detailed in Table 3, demonstrating their high carbohydrate content (Ali et al. 2022). Kharak dates, which are harvested in an unripe stage, exhibit lower initial sugar levels compared to mature dates, as ripening is associated with increased TSS and sugar concentrations, particularly sucrose (Karizaki 2017). This lower sugar content in unripe Kharak dates can contribute to the production of vinegar with naturally mild acidity, an option that may benefit consumers sensitive to high‐acid foods. While the use of unripe fruit to produce vinegar with reduced acidity is not unique to dates, Kharak dates offer a distinctive combination of compounds that support fermentation and yield a high‐quality, lower‐acid vinegar. Additionally, Kharak dates, particularly of the Halileh cultivar, are often considered agricultural byproducts with limited industrial use, mainly consumed locally (Al‐Qurashi 2010). Recent studies suggest that these unripe dates hold potential for value‐added fermentation applications (Hamdi et al. 2023; Sayah et al. 2024), expanding their utility beyond traditional consumption and catering to niche markets with a demand for milder vinegars and unique sensory characteristics.

**TABLE 3:** Main components of Halileh variety of date Kharak.

Characteristics	Content	Characteristics	Content
Moisture (%)	63.2 ± 0.5	Iron (mg/100 g)	2.9 ± 0.1
Total sugar (%)	38 ± 1.2	Phosphorus (mg/100 g)	38 ± 0.8
Reducing sugar (%)	32 ± 1.0	Calcium (mg/100 g)	49 ± 1.5
Fiber (%)	4.1 ± 0.2	Potasium (mg/100 g)	605 ± 10
Protein (%)	1.88 ± 0.1	Ash (%)	2.4 ± 0.1

### 3.2 Identification of Acetic Acid Flora in Vinegar Samples

#### 3.2.1 Characteristics of Colony Appearance

From the purified colonies, 30 isolates were selected for further characterization and assigned unique codes for identification (Table 4). These colonies exhibited morphologies ranging from single to multiple coccobacilli.

**TABLE 4:** Isolates used in PCR.

Isolate number	Isolate	Isolate code	Acidity	pH
1	A	FR11	0.7	6.68
2	B	FR12	0.6	6.70
3	C	FR13	0.7	6.67
4	D	FR21	2.2	4.96
5	E	FR22	2.1	4.92
6	F	FR31	0.7	6.66
7	G	FR32	1.2	5.52
8	H	FR41	1.6	5.38
9	I	FR42	0.5	6.80
10	J	FR43	2.2	4.96
11	K	FR44	1.2	6
12	M	FR51	1	6.16
13	N	FR51	0.7	6.55
14	P	FR53	2	5
15	Q	FR54	0.9	6.26
16	R	FR55	0.9	6.35
17	S	FR61	2.3	4.90
18	T	FR62	0.6	6.74
19	U	FR63	0.6	6.74
20	V	FR71	1.7	5.1
21	W	FR72	0.6	6.70
22	X	FR73	0.8	6.30
23	Y	FR81	0.8	6.32
24	Z	FR82	0.7	6.74
25	AA	FR83	0.7	6.75
26	BB	FR91	0.7	6.70
27	CC	FR92	0.8	6.37
28	DD	FR93	0.7	6.55
29	EE	FR101	0.6	6.73
30	FF	FR102	0.7	6.62

#### 3.2.2 Confirmation of Bacterial Acetic Acid Production

In addition to the observed IZs around the colonies on the plates, further tests were conducted to confirm their characteristics. Gram staining revealed a range of results, with most isolates being Gram‐negative, although some exhibited Gram‐positive characteristics. Additionally, the catalase test produced positive results, which supports the classification of the isolates as AAB.

#### 3.2.3 Identification of Isolates at Genus and Species Levels

**FIGURE 3:**
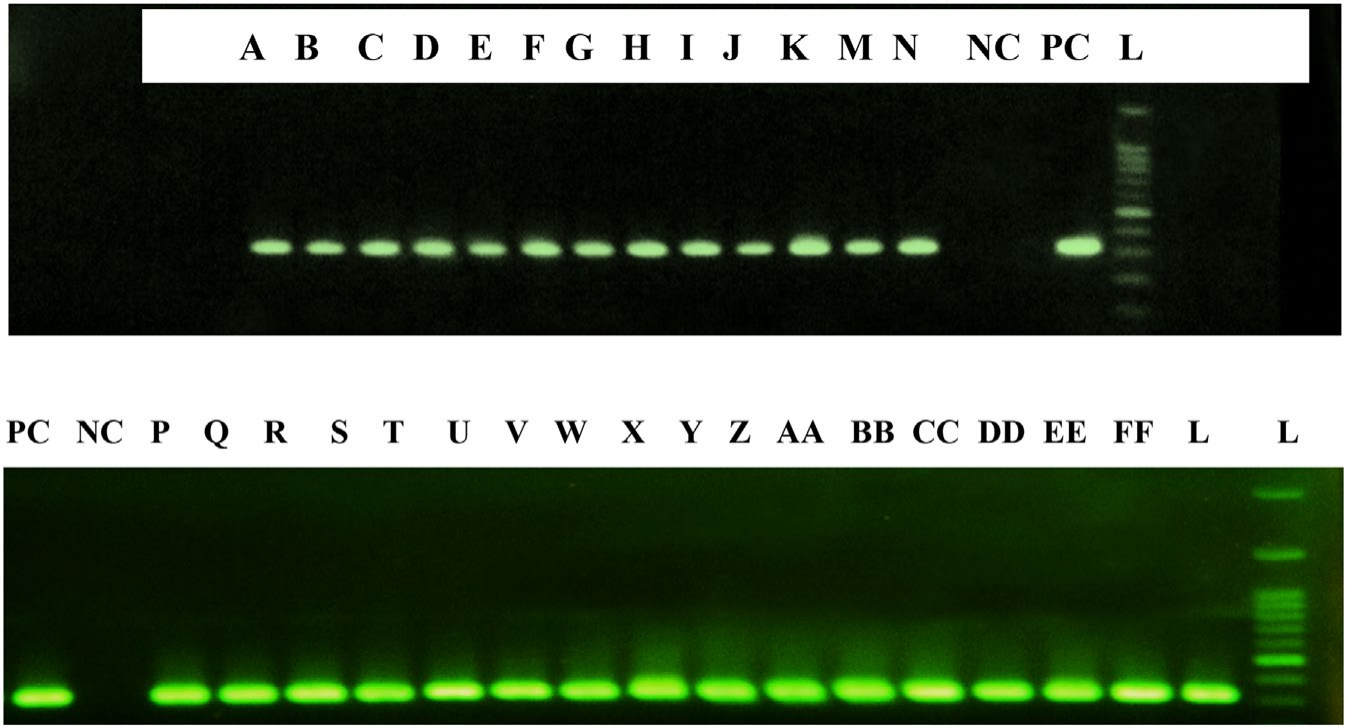
Gel electrophoresis image of PCR products. From right to left: L: DNA ladder, PC, Positive control; NC, Negative control; A to FF: Isolates.

Due to limitations in culture‐based and biochemical methods for genus and species determination, isolates were subjected to molecular testing following purification via streak plating. After activation on GYC agar and subsequent transfer to GY liquid medium, bacterial DNA was isolated using a DNA extraction kit. DNA from 30 selected isolates was then used for PCR amplification targeting the 16S rRNA gene specific to AAB (Lopez et al. 2003; Sengun et al. 2022). Gel electrophoresis of the PCR products (Figure 3) consistently revealed a band size of 385 bp, confirming both the size and quality of the extracted DNA.

### 3.3 Selection of Acetic Acid Bacterial Isolates

To identify superior isolates, we evaluated growth rate, final acidity, pH, and alcohol consumption for all 30 isolates (Table 4). The initial acidity of the medium before fermentation was approximately 0.2% (w/v). Final acidity values, presented in Table 5, indicate the total AA concentration achieved by each isolate after fermentation. From this assessment, 12 isolates showing the highest growth rates, efficient alcohol consumption, significant acid production, and lower final pH were selected for further comparison (Table 5). Among these, isolates FR22, FR43, and FR61 demonstrated superior performance in these criteria and were chosen for subsequent research stages. Following the PCR analysis, products from these isolates were sequenced and matched with sequences in the NCBI database for identification.

**TABLE 5:** Growth, alcohol consumption, reduction efficiency, final acidity, and pH of acetic acid bacterial isolates after 15‐day fermentation in 30% date syrup (initial alcohol: 4.2 g/100 mL).

Isolate numbers	Isolate	Species	Log CFU/mL	Initial alcohol (g/100 mL)	Final alcohol (g/100 mL)	Reduction (%)	Final acidity (% w/v)	Final pH
4	FR21	*A. aceti*	5.48	4.2	0.85	80 ± 2	2.0 ± 0.1	4.8 ± 0.1
5	**FR22**	***A. pasteurianus***	**5.95**	**4.2**	**0.70**	**83 ± 1**	**2.3 ± 0.1**	**4.8 ± 0.1**
7	FR32	*A. tropicalis*	5.70	4.2	0.90	79 ± 2	2.0 ± 0.1	4.9 ± 0.1
8	FR41	*A. aceti*	5.15	4.2	0.90	79 ± 2	2.0 ± 0.1	4.8 ± 0.1
10	**FR43**	***A. aceti***	**5.93**	**4.2**	**0.88**	**79 ± 2**	**2.0 ± 0.1**	**4.7 ± 0.1**
11	FR44	*A. tropicalis*	4.08	4.2	0.87	79 ± 2	2.0 ± 0.1	4.9 ± 0.1
12	FR51	*A. pasteurianus*	4.00	4.2	0.84	80 ± 2	1.3 ± 0.1	4.8 ± 0.1
14	FR53	*A. tropicalis*	4.15	4.2	5.20	—	0.7 ± 0.1	6.1 ± 0.1
15	FR54	*A. pasteurianus*	4.68	4.2	5.70	—	0.7 ± 0.1	6.1 ± 0.1
16	FR55	*A. aceti*	5.00	4.2	1.75	58 ± 3	1.2 ± 0.1	5.6 ± 0.1
17	**FR61**	***A. tropicalis***	**6.23**	**4.2**	**0.80**	**81 ± 1**	**2.2 ± 0.1**	**4.8 ± 0.1**
20	FR71	*A. pasteurianus*	5.08	4.2	0.80	81 ± 1	2.0 ± 0.1	5.1 ± 0.1

*Note:* Data are mean ± SD (*n* = 3). Bold rows indicate key isolates (FR22, FR43, FR61) selected for detailed comparison in the text. Alcohol reduction (%) = [(Initial − Final)/Initial] × 100. Isolates with final alcohol > 5 g/100 mL were excluded from reduction calculation due to incomplete fermentation. Selection thresholds: final count ≥ 6.0 log CFU/mL, alcohol reduction ≥ 80%, acidity ≥ 2.0% w/v, pH drop ≥ 1.0 unit.

### 3.4 Vinegar Production Results

#### 3.4.1 Changes in the Growth of *Saccharomyces cerevisiae*

Initially, within the first 24 h after yeast inoculation, no significant growth occurred in all date concentrations (20% and 40%) or minimal growth was observed in 30%. This period corresponds to the lag phase, during which yeast adapts to its environment. Subsequently, a logarithmic increase in yeast cell numbers was observed across all concentrations, reaching peak levels. Notably, at 20% (Treatment A), significant growth differences were observed compared to the other treatments. In 30% (Treatment B), no significant variance was seen with the other treatments (Tanamool et al. 2020). Conversely, at 40% (Treatment C), initial growth differences were significant but did not persist in the subsequent days of the growth phase. This growth pattern persisted for 48 h before yeast cell numbers stabilized. Following this, from the 4th day onwards, concurrent with the onset of acetic fermentation and AAB growth, yeast cell counts declined steadily until reaching zero on the final day (Li et al. 2023). This decline aligns with Krusong and Vichitraka (2010), who reported yeast death when AAB reached 6–8 log CFU/mL and acetic acid exceeded 2% in simultaneous pineapple vinegar fermentation, indicating antagonism via acetic acid toxicity, consistent with our sequential process.

As indicated in Table 6, yeast growth follows a clear pattern of lag phase, logarithmic growth, and peak levels before a decline, with significant growth differences observed at 20% (Treatment A) compared to the other treatments. Similarly, interactions between yeast and AAB were observed during pineapple vinegar production, noting that AAB's proliferation inhibits yeast growth (Krusong and Vichitraka 2010). AA production reduces yeast cell mass and inhibits growth rate. Cytometric analysis of yeast cell physiological changes during vinegar production, using the fluorescent marker diacetate carboxy‐fluorescein (cFDA), indicated intact cell membranes in living cells. Dead cell identification involved the penetration of the fluorescent dye Propidium Iodide into cell DNA, resulting in an orange color. Moreover, exposure to high AA concentrations leads to cell death during the stationary phase. Electrophoresis of chromosomal DNA from deceased cells in this phase revealed fragmentation and significant chromatin degradation (Srikanlayanukul and Sillapawattana 2022).

**TABLE 6:** Yeast growth data.

Day	20% date (treatment A)	30% date (treatment B)	40% date (treatment C)
1	2.5 (lag phase)	2.3 (lag phase)	2.6 (lag phase)
2	4.0 (log phase)	4.1 (log phase)	4.2 (log phase)
3	6.2 (peak)	6.0 (peak)	6.1 (peak)
4	5.8 (decline starts)	5.5 (decline starts)	5.6 (decline starts)
5	5.0	5.2	5.1
6	4.2	4.5	4.3
7	3.0	3.2	3.1

*Note:* Model preference: Rational (*R* ^2^ ≥ 0.98, RMSE ≤ 0.12; pH/TSS/alcohol); Weibull (*R* ^2^ ≥ 0.97, RMSE ≤ 0.15; acidity).

Yeast is renowned for its ability to ferment hexose sugars into ethanol. Yeast growth during vinegar production undergoes three distinct phases: growth, stress, and cell death. Stress arises primarily from ethanol production and culminates in yeast cell demise. The formation of “mother vinegar” denotes the growth phase, while the stationary phase signifies alcohol oxidation to AA by bacteria, culminating in “mother vinegar” sedimentation, signifying the completion of acetic fermentation. These observations align with reports emphasizing optimal cell mass under high alcohol and AA concentrations (Zhang et al. 2024). Alcohol production imposes stress on yeast cells, precipitating their clumping, which occurs as the growth phase ends, coinciding with alcohol's peak production. Clumping serves as a stress response and defense mechanism against high alcohol concentrations. Acetaldehyde, a byproduct of ethanol biological oxidation by *Acetobacter*, chiefly affects yeast enzymatic activity (Matsumoto et al. 2021).

AA alters both extracellular and intracellular yeast pH. With low extracellular pH, AA permeates cell membranes. Upon entering cells, AA can dissociate into H^+^ and CH_3_COO^−^ due to the near‐neutral pH of the cytoplasm, contributing to intracellular acidification and lowering the internal pH. AA's toxicity surpasses that of ethanol by 30‐fold. Cell death from AA or ethanol exposure depends on growth stage, initial conditions, and laboratory settings. Cultivation variables such as temperature, pH, oxygen availability, nutrient access, and glucose concentration significantly influence cell death rates under AA stress (Paul et al. 2024).

#### 3.4.2 Changes in the Growth of Acetic Acid Bacteria

**FIGURE 4:**
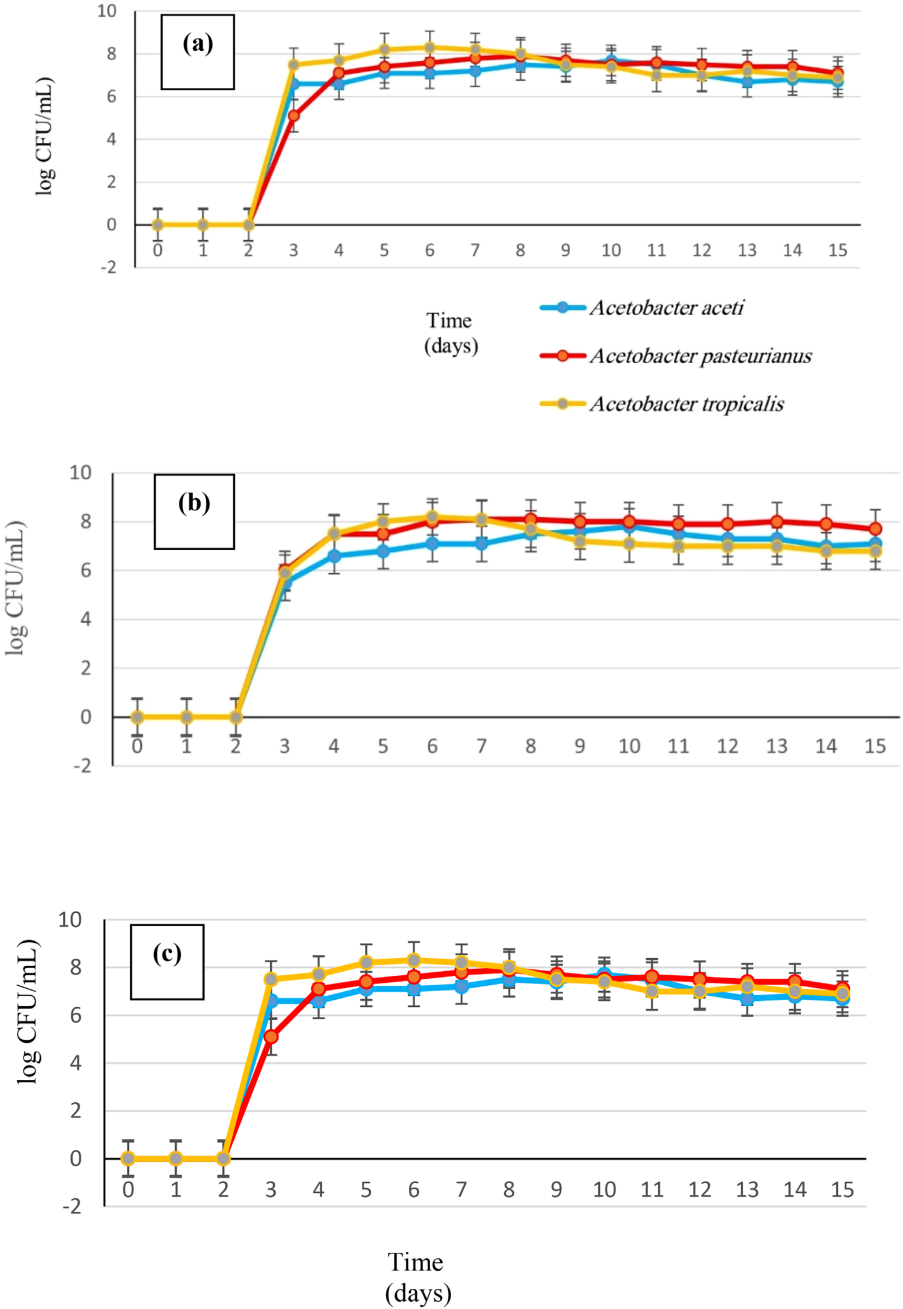
Cell changes of acetic acid bacteria in the alcoholic extract obtained from the fermentation of date over 15 days. (a) 20%, (b) 30%, (c) 40% date.

Several factors influence AAB growth and survival, including ethanol and AA concentrations, oxygen levels, temperature, and nutrient availability. AAB exhibit robust growth below 10 g/L AA, particularly when ethanol levels are low, with growth continuing until approximately 20 g/L AA concentration. Higher AA (~50 g/L) severely restricts bacterial growth. Oxygen availability significantly impacts AAB growth during vinegar acetic fermentation, consistent with their aerobic nature (Nassir and Al‐Sahlany 2021). Figure 4a depicts changes in three isolates of *Acetobacter pasteurianus* , *A. aceti* , and *A. tropicalis* in 20% date alcoholic extract fermentation. Initially, all isolates exhibited significant growth within the first 24 h. By the second day, only *A. tropicalis* showed significant growth differences. *A. tropicalis* maintained superior growth rates over *A. pasteurianus* and *A. aceti* , which continued to increase until the 4th and 6th days post‐inoculation, respectively, followed by a logarithmic decline. *A. pasteurianus* notably sustained growth throughout, making it the preferred isolate for AA production in this concentration. As shown in Table 5, key isolate FR22 ( *A. pasteurianus* ) achieved the highest final acidity (2.3% ± 0.1% w/v) and alcohol reduction (83% ± 1%), with stable pH (4.8 ± 0.1), confirming its superiority.

Its resilience aligns with Boondaeng et al. (2025), who reported *A. pasteurianus* outperforming other AAB in Agave‐pineapple blends, though their simultaneous approach and nutrient supplementation (DAP/MgSO_4_) yielded 5.5% acetic acid. Our sequential, single‐substrate design at 30% date syrup prioritized mild acidity (2.3%) and sustained *A. pasteurianus* viability without additives, ideal for dietary vinegar (Boondaeng et al. 2025).

Key isolate FR22 ( *A. pasteurianus* ) achieved final acidity of 2.3% ± 0.1% (w/v), pH = 4.8 ± 0.1, and 85% alcohol reduction (from 4.2 to 0.63 g/100 mL) after 15 days, outperforming FR43 ( *A. aceti* : 2.0% acidity, pH 4.7, 82% reduction) and FR61 ( *A. tropicalis* : 2.2% acidity, pH 4.8, 80% reduction) (Table 5). These metrics are lower than industrial benchmarks (7.2% acidity, 90%+ reduction in Tanamool et al. 2020; 5.5% acidity in Boondaeng et al. 2025) but ideal for mild dietary vinegar.

In the 30% alcoholic extract fermentation (Figure 4b), *A. aceti* initially exhibited significant growth within 24 h, with no significant differences observed between isolates by day 2. Contrary to the 20% extract, all three isolates displayed similar growth patterns throughout the fermentation process, with significant differences in *A. pasteurianus* cell numbers noted on the final day. Consequently, all three isolates are viable options for this process due to their comparable growth rates. Figure 4c illustrates changes in three isolates during 40% date alcoholic extract fermentation. Initial *A. pasteurianus* cell counts were lower upon inoculation but displayed noticeable growth within 24 h. In contrast, *A. aceti* and *A. tropicalis* exhibited slower initial growth, potentially contributing to *A. pasteurianus'* higher growth rate. Significant differences were observed among all treatments at this stage. At the end of the process, *A. pasteurianus* cell counts significantly exceeded those of *A. aceti* . Calculation of growth rate revealed *A. pasteurianus'* superior stability and performance compared to *A. aceti* (7.55%) and *A. tropicalis* (8.56%), respectively (Ouattara et al. 2023).

In their study, Krusong and Vichitraka (2010) observed the growth trajectory of AAB during vinegar fermentation involving yeast and AAB. They reported that following inoculation under aerobic conditions, AAB adapted to their environment, exhibiting growth at a rate equivalent to 2 Log cycles. Throughout the fermentation process, the cell count remained relatively stable without significant changes (Ye et al. 2024). AAB demonstrate three distinct growth phases in environments containing ethanol. Although growth patterns vary among species, these phases can be broadly categorized as: (i) rapid oxidation of ethanol to AA releases energy into the surrounding environment (ethanol oxidation phase); (ii) a lag phase ensues during which the cell growth rate diminishes or cells enter a resting state (lag phase); (iii) a second exponential phase (acetate oxidation phase) occurs wherein AA is metabolized by ADH and ALDH enzymes in the cytoplasm to generate energy and carbon. AA accumulates during the ethanol oxidation and resting phases and is subsequently decomposed into water and carbon dioxide during acetate oxidation when ethanol is depleted (Klawpiyapamornkun et al. 2015). Our sequential design minimized the acetate over‐oxidation phase, preserving acetic acid yield, unlike simultaneous systems (Krusong and Vichitraka 2010; Tanamool et al. 2020), where AAB entered over‐oxidation earlier due to prolonged yeast‐AAB co‐existence.

Strains well‐suited for vinegar production exhibit minimal consumption of AA during the initial logarithmic phase and demonstrate tolerance to high AA concentrations during the resting phase (Ye et al. 2024). Across all three tested concentrations (20%, 30%, and 40%), Figure 4 depicts a reduction in cell growth during the stationary phase, with *A. pasteurianus* isolates exhibiting lesser decline compared to *A. aceti* and *A. tropicalis* isolates. This resilience to AA suggests that *A. pasteurianus* is a favorable isolate for vinegar production. Different strains exhibit varying degrees of AA resistance during fermentation, influenced by cell concentration, culture conditions, and the interval between strain extraction and use. The impact of AA on AAB growth is contingent upon substrate (ethanol) and product (AA) concentrations, as well as growth conditions. Research indicates that a threshold ethanol concentration > 3% delays the onset of secondary growth phases, with 4% and 5% ethanol negating the lag phase altogether, thereby halting acetate oxidation. Additionally, initial ethanol levels significantly affect the duration of the initial growth phase, with most AAB capable of growth in the presence of up to 5% ethanol in fermentation media, albeit few strains maintaining this capability at 10% ethanol levels (Cui et al. 2024). In acetic vinegar preparation and fermentation, alcohol concentrations rarely exceed 10%, thus minimizing constraints on AAB growth. High concentrations of ethanol and AA in the environment have been shown to decrease cell mass growth, a phenomenon rarely encountered as ethanol concentrations seldom exceed 10%.

#### 3.4.3 Microbial Growth Modeling

Tables 7 and 8 present the modeling of yeast and AAB growth in various date concentrations using Gaussian and Gompertz models, respectively. To accurately represent the growth behavior, we based our modeling on experimental data collected from cultures grown in 20%, 30%, and 40% date concentrations. For each concentration, we measured initial sugar content and growth rates, focusing on the correlation between sugar availability, medium viscosity, and microbial growth. The yeast growth model (Table 7) reveals that a 20% date concentration results in the highest R^2^ (0.99) and the lowest error rate (0.26). This indicates optimal yeast proliferation at this concentration, likely due to the availability of sugar (11.2% at 20% date concentration). As the sugar content increases with higher concentrations (14.28% for 30% and 17.92% for 40%), medium viscosity also rises, which appears to restrict yeast access to sugars, thereby inhibiting growth. This interpretation aligns with previous findings that high viscosity can impede nutrient transport to microbial cells (Zheng et al. 2022).

**TABLE 7:** Yeast growth modeling during alcoholic fermentation of date vinegar.

Model	Equation	Concentration	*R* ^2^	Error(s)
Gaussion	$y=ae^{-{\left(x-b\right)}^{2}/2c^{2}}$	20%	0.99	0.26
		30%	0.98	0.34
		40%	0.99	0.30

**TABLE 8:** Modeling the growth of acetic acid bacteria during acetic fermentation of date vinegar.

Model	Equation	Concentration	Strain	Correlation coefficient (*R* ^2^)	Error(s)
Gompertz	$y=ae^{-e^{b-\mathrm{cx}}}$	20%	*A. pasteurianus*	0.95	0.23
			*A. aceti*	0.86	0.42
			*A. tropicalis*	0.56	0.53
		30%	*A. pasteurianus*	0.96	0.15
			*A. aceti*	0.92	0.24
			*A. tropicalis*	0.64	0.53
		40%	*A. pasteurianus*	0.96	0.20
			*A. aceti*	0.52	0.31
			*A. tropicalis*	0.49	0.53

In Table 8, we model the growth of isolated AAB strains, including *A. pasteurianus* , *A. aceti* , and *A. tropicalis* , in alcoholic extracts. The data show that *A. pasteurianus* demonstrates the most robust growth across all concentrations, particularly at 30% date, where it achieved the highest R^2^ (0.96) and lowest error (0.15). This suggests that *A. pasteurianus* thrives in these conditions, likely due to its tolerance for moderate alcohol levels. Conversely, *A. tropicalis* exhibited limited growth across all concentrations, with lower R^2^ and higher error rates, indicating its sensitivity to the fermentation conditions used in this study.

### 3.5 Modeling the Factors Affecting the Fermentation

In this stage, mathematical models were employed to analyze the factors influencing the fermentation process of DV, with particular focus on *A. pasteurianus* as the primary bacterial strain. Factors modeled included TSS, pH, acidity, and alcohol content. The goal was to optimize fermentation conditions by identifying the most effective date concentration and fermentation parameters for the growth of *A. pasteurianus* . To model the changes in Brix across different date concentrations, we evaluated multiple models and found that the Michaelis–Menten Function (MMF) model provided the best fit for 20% date extract (Tables S1 and S2, Supporting Information). For 30% and 40% date levels, the Rational Function model showed a stronger correlation (*R* ^2^ ≥ 0.98, RMSE ≤ 0.12). However, differences between the models were minimal at these higher concentrations. For pH changes, the Rational model emerged as the most consistent across all concentrations, demonstrating a strong correlation with observed pH variations (*R* ^2^ ≥ 0.98, RMSE ≤ 0.08). While other models provided reasonable fits for the 20% date extract, the Rational model was particularly effective for 30% and 40% levels due to its ability to capture non‐monotonic asymptotic trends (Tables S3 and S4, Supporting Information).

For modeling acidity changes during fermentation, both the Logistic and Weibull models were evaluated. Although both models performed well, the Weibull model showed slightly better accuracy, especially for 20% and 30% date extracts (*R* ^2^ ≥ 0.97, RMSE ≤ 0.15). The Weibull model was preferred for its flexible shape parameters (*α*, *β*) enabling superior fit to asymmetrical sigmoidal acidity accumulation. Therefore, we recommend using the Weibull model for precise acidity predictions (Tables S5 and S6, Supporting Information). For alcohol content, the Rational model again proved to be the most effective, with high *R* ^2^ for 30% and 40% date concentrations (*R* ^2^ ≥ 0.98, RMSE ≤ 0.11). Although the Sinusoidal Fit and Gaussian models were also applicable, the Rational model consistently provided the most reliable results due to robust handling of plateau and decay phases (Tables S7 and S8, Supporting Information).

The suitability of models across different fermentation parameters showed that the MMF model was most appropriate for predicting Brix changes in 20% date, while the Rational model provided a better fit for 30% and 40% concentrations. For pH, the Rational model demonstrated reliable accuracy across all concentrations. When modeling acidity, the Weibull model proved slightly more accurate, particularly for 20% and 30% date extracts. Finally, for alcohol content, the Rational model showed the highest accuracy, especially at the 30% date concentration. Overall, the Rational model was found to be the most versatile and accurate for modeling various fermentation parameters due to highest R^2^ and lowest RMSE across non‐sigmoidal datasets. It was especially effective for predicting Brix, pH, and alcohol content changes. For acidity, the Weibull model provided slightly better performance via flexible sigmoidal kinetics. Through these models, a date concentration of 30% was identified as optimal for achieving favorable fermentation results, as confirmed by the performance of the Rational model across multiple parameters. These findings align with prior research, which has shown that moderate sugar and alcohol concentrations support optimal microbial growth during fermentation (Salakkam et al. 2023).

These predictive models enable industrial‐scale production of high‐quality date vinegar from unripe Kharak byproducts, promoting sustainability through waste valorization, process automation, and consistent mild‐acidity output suitable for dietary and functional food markets.

## 4 Conclusions

This study demonstrates that date vinegar (DV) production from unripe Kharak dates is a sustainable approach to utilizing agricultural byproducts. Among the acetic acid bacteria (AAB) evaluated, *Acetobacter pasteurianus* exhibited superior performance in fermenting date syrup. Suitable AAB isolates were selected based on high acid production (≥ 2.0% acidity), fermentation efficiency (≥ 80% alcohol reduction), and stability across 20%–40% date syrup concentrations. *A. pasteurianus* achieved a final acetic acid concentration of 4.98% (w/v) and a pH of 3.2 at 30% date syrup concentration, outperforming other strains in both yield and consistency. The 30% date syrup concentration proved optimal for producing high‐quality, low‐acidity vinegar suitable for dietary needs. We applied Gaussian, Gompertz, Rational, Sinusoidal Fit, and Logistic models to analyze fermentation kinetics. These models provided predictive insights, optimizing key variables like temperature and starter concentration. By integrating microbial characterization with advanced modeling, this research enhances the understanding of DV fermentation dynamics. The use of Kharak dates not only reduces agricultural waste but also supports the production of a value‐added product with potential nutritional and economic benefits. This work establishes a scalable framework for sustainable DV production, paving the way for industrial applications and further sensory and nutritional studies.

## Author Contributions

**Fatemeh Rasi:** data curation (equal), formal analysis (equal), investigation (equal), writing – original draft (equal). **Morteza Khomeiri:** formal analysis (equal), methodology (equal), project administration (equal), supervision (equal), validation (equal), visualization (equal). **Seid Mahdi Jafari:** conceptualization (equal), funding acquisition (equal), project administration (lead), supervision (lead), validation (equal), writing – review and editing (lead). **Mahdi Kashaninejad:** formal analysis (equal), methodology (equal), validation (equal). **Alireza Sadeghi:** formal analysis (equal), methodology (equal), validation (equal).

## Funding

The authors did not receive any specific funding for this study.

## Conflicts of Interest

The authors declare no conflicts of interest.

## Supporting information


**Data S1:** fsn371605‐sup‐0001‐Tables.docx.

## Data Availability

Research data are not shared.

## References

[fsn371605-bib-0001] Adedayo, M. R. , and J. Mohammed . 2024. “Isolation, Identification, and Screening of Bacteria From Yam Waste for Amylase Production Ability.” International Journal of Applied Chemical and Biological Sciences 5, no. 1: 12–21.

[fsn371605-bib-0002] Ali, Z. , J. Li , Y. Zhang , N. Naeem , S. Younas , and F. Javeed . 2022. “Dates (Phoenix Dactylifera) and Date Vinegar: Preventive Role Against Various Diseases and Related In Vivo Mechanisms.” Food Reviews International 38, no. 4: 480–507.

[fsn371605-bib-0003] Ali, Z. , H. Ma , M. T. Rashid , A. Wali , and S. Younas . 2019. “Preliminary Study to Evaluate the Phytochemicals and Physiochemical Properties in Red and Black Date's Vinegar.” Food Science & Nutrition 7, no. 6: 1976–1985. 31289645 10.1002/fsn3.1009 PMC6593385

[fsn371605-bib-0005] Ali, Z. , H. Ma , A. Wali , I. Ayim , and M. N. Sharif . 2019. “Daily Date Vinegar Consumption Improves Hyperlipidemia, β‐Carotenoid and Inflammatory Biomarkers in Mildly Hypercholesterolemic Adults.” Journal of Herbal Medicine 17: 100265.

[fsn371605-bib-0006] Al‐Qaisi, A. , M. Alrosan , A. M. Almajwal , et al. 2024. “Evaluation of Structure, Quality, Physicochemical Properties, and Phenolics Content of Pea Proteins: A Novel Strategy Through the Incorporation of Fermentation.” Journal of Food Science 89, no. 3: 1517–1530. 38317408 10.1111/1750-3841.16946

[fsn371605-bib-0007] Al‐Qurashi, A. D. 2010. “Physico‐Chemical Changes During Development and Ripening Of'helali'date Palm Fruit.” Journal of Food, Agriculture & Environment 8, no. 2: 404–408.

[fsn371605-bib-0008] Boondaeng, A. , C. Trakunjae , and N. Niyomvong . 2025. “Optimization of Agave‐Based Pineapple Vinegar Fermentation Through Sequential Fermentation and Chemical Characterization.” Applied Food Research 5, no. 1: 100826.

[fsn371605-bib-0009] Budiarti, L. , A. Yasmina , P. Nurikwan , M. Prayudi , M. Firisa , and Y. Kangsudarmanto . 2022. “Antibacterial Activity of Infused Peel of Kaffir Lime, Manurun Banana, and Pineapple Against the Number of *Staphylococcus aureus* and *Escherichia coli* Colonies.” Paper presented at the IOP Conference Series: Earth and Environmental Science.

[fsn371605-bib-0010] Chakraborty, K. , S. K. Saha , U. Raychaudhuri , and R. Chakraborty . 2018. “Vinegar Production From Vegetable Waste: Optimization of Physical Condition and Kinetic Modeling of Fermentation Process.” Indian Journal of Chemical Technology (IJCT) 24, no. 5: 508–516.

[fsn371605-bib-0011] Choudhary, M. , S. Joshi , S. S. Bhagyawant , and N. Srivastava . 2018. “Advances in Fermentation Technology: Principle and Their Relevant Applications.” In Principles and Applications of Fermentation Technology, 53–63. Wiley.

[fsn371605-bib-0012] Cui, M. , M. Wang , H. Sun , et al. 2024. “Identifying and Characterization of Novel Broad‐Spectrum Bacteriocins From the Shanxi Aged Vinegar Microbiome: Machine Learning, Molecular Simulation, and Activity Validation.” International Journal of Biological Macromolecules 270: 132272. 38734334 10.1016/j.ijbiomac.2024.132272

[fsn371605-bib-0013] De Vero, L. , E. Gala , M. Gullo , L. Solieri , S. Landi , and P. Giudici . 2006. “Application of Denaturing Gradient Gel Electrophoresis (DGGE) Analysis to Evaluate Acetic Acid Bacteria in Traditional Balsamic Vinegar.” Food Microbiology 23, no. 8: 809–813. 16943087 10.1016/j.fm.2006.01.006

[fsn371605-bib-0014] Gerard, L. M. , C. V. Davies , C. A. Soldá , M. B. Corrado , and M. V. Fernández . 2020. “Application of Molecular Methods for the Identification of Acetic Acid Bacteria Isolated From Blueberries and Citrus Fruits.” Microbiology and Biotechnology Letters 48: 193–204.

[fsn371605-bib-0015] Ghosh, S. , R. Chakraborty , G. Chatterjee , and U. Raychaudhuri . 2012. “Study on Fermentation Conditions of Palm Juice Vinegar by Response Surface Methodology and Development of a Kinetic Model.” Brazilian Journal of Chemical Engineering 29: 461–472.

[fsn371605-bib-0016] Hamdi, A. , I. Viera‐Alcaide , S. Costa , et al. 2023. “A Sustainable Approach for the Valorization of Underutilized Date Fruits.” Molecules 28, no. 15: 5807. 37570777 10.3390/molecules28155807 PMC10420846

[fsn371605-bib-0017] Hegazy, A. G. , M. Melebari , F. M. Al Guthami , et al. 2024. “Physicochemical, Antimicrobial and Bioactive Properties of Date Vinegar.” Egyptian Journal of Veterinary Science: 1–9. 10.21608/EJVS.2024.299982.2208.

[fsn371605-bib-0018] Joshi, V. K. , R. Sharma , V. Kumar , and D. Joshi . 2019. “Optimization of a Process for Preparation of Base Wine for Cider Vinegar Production.” Proceedings of the National Academy of Sciences, India Section B: Biological Sciences 89: 1007–1016.

[fsn371605-bib-0019] Karizaki, V. M. 2017. “Iranian Dates and Ethnic Date‐Based Products.” Journal of Ethnic Foods 4, no. 3: 204–209.

[fsn371605-bib-0020] Klawpiyapamornkun, T. , S. Bovonsombut , and S. Bovonsombut . 2015. “Isolation and Characterization of Acetic Acid Bacteria From Fruits and Fermented Fruit Juices for Vinegar Production.” Food and Applied Bioscience Journal 3, no. 1: 30–38.

[fsn371605-bib-0021] Kourouma, M. C. , M. Mbengue , A. Thioye , and C. T. Kane . 2023. “Response Surface Methodology as an Approach for Optimization of Vinegar Fermentation Conditions Using Three Different Thermotolerant Acetic Acid Bacteria.” Food and Nutrition Sciences 14, no. 7: 638–656.

[fsn371605-bib-0022] Krusong, W. , and A. Vichitraka . 2010. “An Investigation of Simultaneous Pineapple Vinegar Fermentation Interaction Between Acetic Acid Bacteria and Yeast.” Asian Journal of Food and Agro‐Industry 3, no. 1: 192–203.

[fsn371605-bib-0023] Kubizniaková, P. , L. Kyselová , M. Brožová , K. Hanzalíková , and D. Matoulková . 2021. “The Role of Acetic Acid Bacteria in Brewing and Their Detection in Operation.” Kvasny Prumysl 67, no. 5: 511–522.

[fsn371605-bib-0024] Li, S. , P. Li , F. Feng , and L.‐X. Luo . 2015. “Microbial Diversity and Their Roles in the Vinegar Fermentation Process.” Applied Microbiology and Biotechnology 99: 4997–5024. 25971198 10.1007/s00253-015-6659-1

[fsn371605-bib-0025] Li, Y.‐N. , Y. Luo , Z.‐M. Lu , et al. 2023. “Metabolomic Analysis of the Effects of a Mixed Culture of *Saccharomyces cerevisiae* and *Lactiplantibacillus plantarum* on the Physicochemical and Quality Characteristics of Apple Cider Vinegar.” Frontiers in Nutrition 10: 1142517. 36998906 10.3389/fnut.2023.1142517 PMC10043408

[fsn371605-bib-0026] Lopez, I. , F. Ruiz‐Larrea , L. Cocolin , et al. 2003. “Design and Evaluation of PCR Primers for Analysis of Bacterial Populations in Wine by Denaturing Gradient Gel Electrophoresis.” Applied and Environmental Microbiology 69, no. 11: 6801–6807. 14602643 10.1128/AEM.69.11.6801-6807.2003 PMC262258

[fsn371605-bib-0027] Matsumoto, N. , N. Osumi , M. Matsutani , et al. 2021. “Thermal Adaptation of Acetic Acid Bacteria for Practical High‐Temperature Vinegar Fermentation.” Bioscience, Biotechnology, and Biochemistry 85, no. 5: 1243–1251. 33686416 10.1093/bbb/zbab009

[fsn371605-bib-0028] Nassir, T. H. , and S. Al‐Sahlany . 2021. “Bioethanol Production From Agricultural Wastes by Zymomonas Mobilis and Used in Vinegar Production.” Journal of Microbiology, Biotechnology and Food Sciences 11, no. 3: e3709–e3709.

[fsn371605-bib-0029] Nosratabadi, L. , H.‐R. Kavousi , R. Hajimohammadi‐Farimani , M. Balvardi , and S. Yousefian . 2024. “Estamaran Date Vinegar: Chemical and Microbial Dynamics During Fermentation.” Brazilian Journal of Microbiology 55, no. 2: 1265–1277. 38696037 10.1007/s42770-024-01354-6 PMC11153425

[fsn371605-bib-0030] Nyuykongi, M. , W. A. Ambindei , N. P. Ndasi , N. M. Ngwabie , M. B. Ngassoum , and N. E. Jong . 2023. “Effect of Acid Pre‐Treatment and Two‐Stage Oxygen‐Assisted Fermentation on the Production of Vinegar From Lignocellulose Biomass Peel of Pineapple. Figure Legends Figure, 1.”

[fsn371605-bib-0031] Ouattara, A. , M. K. Somda , C. A. Ouattara , et al. 2023. “Production of Vinegar Mango Using *Acetobacter tropicalis* CRSBAN‐BVA1 and CRSBAN‐BVK2 Isolated From Burkina Faso.” Food and Nutrition Sciences 14, no. 1: 26–37.

[fsn371605-bib-0032] Paul, S. , J. Wartu , and A. Orukotan . 2024. “Production of Vinegar From Waste Fruits Using Acetobacter Species.” IFE Journal of Science 26, no. 1: 179–200.

[fsn371605-bib-0033] Perumpuli, P. , and D. Dilrukshi . 2022. “Vinegar: A Functional Ingredient for Human Health.” International Food Research Journal 29, no. 5: 959–974.

[fsn371605-bib-0500] Ramadhani, S. I. , S. Prabaningtyas , A. Witjoro , R. T. Saptawati , and A. Rodiansyah . 2020. “Quantitative Assay of Indole Acetic Acid‐Producing Bacteria Isolated from Several Lakes in East Java, Indonesia.” Biodiversitas 21, no. 11: 5448–5454. 10.13057/biodiv/d211153.

[fsn371605-bib-0034] Román‐Camacho, J. J. , I. García‐García , I. M. Santos‐Dueñas , T. García‐Martínez , and J. C. Mauricio . 2023. “Latest Trends in Industrial Vinegar Production and the Role of Acetic Acid Bacteria: Classification, Metabolism, and Applications—A Comprehensive Review.” Food 12, no. 19: 3705. 10.3390/foods12193705 PMC10572879 37835358

[fsn371605-bib-0035] Salakkam, A. , N. Phukoetphim , P. Laopaiboon , and L. Laopaiboon . 2023. “Mathematical Modeling of Bioethanol Production From Sweet Sorghum Juice Under High Gravity Fermentation: Applicability of Monod‐Based, Logistic, Modified Gompertz and Weibull Models.” Electronic Journal of Biotechnology 64: 18–26.

[fsn371605-bib-0036] Sayah, I. , C. Gervasi , S. Achour , and T. Gervasi . 2024. “Fermentation Techniques and Biotechnological Applications of Modified Bacterial Cellulose: An up‐To‐Date Overview.” Fermentation 10, no. 2: 100.

[fsn371605-bib-0037] Selvanathan, Y. , and N. Masngut . 2023. “Optimization of Process Factor and Characterization of Vinegar‐Like Beverage Production via Spontaneous Fermentation From Pineapple Peel Waste.” LWT 182: 114818.

[fsn371605-bib-0038] Sengun, I. Y. , G. Kilic , P. Charoenyingcharoen , P. Yukphan , and Y. Yamada . 2022. “Investigation of the Microbiota Associated With Traditionally Produced Fruit Vinegars With Focus on Acetic Acid Bacteria and Lactic Acid Bacteria.” Food Bioscience 47: 101636.

[fsn371605-bib-0039] Song, J. , J. Wang , X. Wang , et al. 2022. “Improving the Acetic Acid Fermentation of *Acetobacter pasteurianus* by Enhancing the Energy Metabolism.” Frontiers in Bioengineering and Biotechnology 10: 815614. 35350179 10.3389/fbioe.2022.815614 PMC8957916

[fsn371605-bib-0040] Soumahoro, S. , H. G. Ouattara , M. Droux , W. Nasser , S. L. Niamke , and S. Reverchon . 2020. “Acetic Acid Bacteria (AAB) Involved in Cocoa Fermentation From Ivory Coast: Species Diversity and Performance in Acetic Acid Production.” Journal of Food Science and Technology 57: 1904–1916. 32327801 10.1007/s13197-019-04226-2 PMC7171043

[fsn371605-bib-0041] Srikanlayanukul, M. , and P. Sillapawattana . 2022. “The Production of Vinegar Cider From Spent Coffee Grounds.” Food and Applied Bioscience Journal 10, no. 3: 1–10.

[fsn371605-bib-0042] Tanamool, V. , M. Chantarangsee , and W. Soemphol . 2020. “Simultaneous Vinegar Fermentation From a Pineapple By‐Product Using the Co‐Inoculation of Yeast and Thermotolerant Acetic Acid Bacteria and Their Physiochemical Properties.” 3 Biotech 10, no. 3: 115. 10.1007/s13205-020-2119-4. PMC7021874 32117676

[fsn371605-bib-0043] Tang, M. , Z. Wang , J. Luo , T. Zhu , F. Song , and H. Chen . 2024. “Preparation, Chemical Profiles, Antioxidative Activities, and Angiotensin‐Converting Enzyme 2 Inhibitory Effect of Date Fruit Vinegar.” Journal of Food Science 89, no. 1: 684–700. 38010752 10.1111/1750-3841.16782

[fsn371605-bib-0044] Tang, R. , Y. Zhu , Y. Yu , S. Cheng , L. Zhou , and K. Wang . 2024. “Optimization and Characterization of a Biphasic Solvent System for Vinegar Residue Pretreatment: Enhanced Fractionation Efficiency, Propionic Acid Production, and Sustainability.” Industrial Crops and Products 222: 119575.

[fsn371605-bib-0045] Tesfaye, W. , M. L. Morales , M. Garcıa‐Parrilla , and A. Troncoso . 2002. “Wine Vinegar: Technology, Authenticity and Quality Evaluation.” Trends in Food Science & Technology 13, no. 1: 12–21.

[fsn371605-bib-0046] Wang, B. , K. Rutherfurd‐Markwick , N. Liu , X.‐X. Zhang , and A. N. Mutukumira . 2024. “Probiotic Potential of Acetic Acid Bacteria Isolated From Kombucha in New Zealand In Vitro.” Microbe 4: 100130.

[fsn371605-bib-0047] Wang, B. , K. Rutherfurd‐Markwick , X.‐X. Zhang , and A. N. Mutukumira . 2022. “Isolation and Characterisation of Dominant Acetic Acid Bacteria and Yeast Isolated From Kombucha Samples at Point of Sale in New Zealand.” Current Research in Food Science 5: 835–844. 35600538 10.1016/j.crfs.2022.04.013 PMC9121233

[fsn371605-bib-0048] Wang, F. , Y. Song , S. K. Vidyarthi , and R. Zhang . 2022. “Physicochemical Properties, and Volatile Compounds of Blackened Jujube Vinegar as Prepared by Optimized Fermentation Process.” International Journal of Food Properties 25, no. 1: 288–304.

[fsn371605-bib-0049] Yang, S. , K. Li , J. Lu , and D. Wu . 2024. “Optimization of Fermentation Conditions and Analysis of the Changes in Flavor Compounds for Lemon Vinegar.” Food Bioscience 62: 105128.

[fsn371605-bib-0050] Ye, X. , Y. Yu , J. Liu , et al. 2024. “Seasonal Environmental Factors Drive Microbial Community Succession and Flavor Quality During Acetic Acid Fermentation of Zhenjiang Aromatic Vinegar.” Frontiers in Microbiology 15: 1442604. 39171262 10.3389/fmicb.2024.1442604 PMC11335490

[fsn371605-bib-0051] Zhang, Z. , Z.‐h. Zhang , R. He , et al. 2024. “Research Advances in Technologies and Mechanisms to Regulate Vinegar Flavor.” Food Chemistry 460: 140783. 39137579 10.1016/j.foodchem.2024.140783

[fsn371605-bib-0052] Zheng, Y. , C. Zhao , X. Li , et al. 2022. “Kinetics of Predominant Microorganisms in the Multi‐Microorganism Solid‐State Fermentation of Cereal Vinegar.” LWT 159: 113209.

