# Supplementary material for: Predictive Modeling of Sequential Fermentation for Sustainable Date Vinegar Production From Unripe Kharak Dates
Source: Food Sci Nutr. 2026 Apr 10;14(4):e71605. doi: 10.1002/fsn3.71605 (PMC13068996; doi:10.1002/fsn3.71605)
Supplement: Supplementary file 1 — Data S1: fsn371605‐sup‐0001‐Tables.docx. [file FSN3-14-e71605-s001.docx]

**Table S1.** Presented models for Brix changes with the growth of *A. pasteurianus* in alcoholic extracts

| **Extract** | **Model** | **Equation** | **Correlation coefficient (R^2^)** | **Error )S(** |
| --- | --- | --- | --- | --- |
| 20% | MMF | $\boldsymbol{y=}\frac{\boldsymbol{ab+}\boldsymbol{cx}^{\boldsymbol{d}}}{\boldsymbol{b+}\boldsymbol{x}^{\boldsymbol{d}}}$ | 0.98 | 0.121 |
|  | Rational Function | $\boldsymbol{y=}\frac{\boldsymbol{a+bx}}{\boldsymbol{1+cx+d}\boldsymbol{x}^{\boldsymbol{2}}}$ | 0.87 | 0.396 |
|  | Gaussion | $\boldsymbol{y=a}\boldsymbol{e}^{\boldsymbol{-(x-b)}^{\boldsymbol{2}}\mathbf{/2}\boldsymbol{c}^{\boldsymbol{2}}}$ | 0.86 | 0.385 |
|  | Quadratic Fit | $\boldsymbol{y=a+bx+}\boldsymbol{cx}^{\boldsymbol{2}}$ | 0.86 | 0.388 |
| 30% | MMF | $\boldsymbol{y=}\frac{\boldsymbol{a+bx}}{\boldsymbol{1+cx+d}\boldsymbol{x}^{\boldsymbol{2}}}$ | 0.98 | 0.101 |
|  | Rational Function | $\boldsymbol{y=a+bx+}\boldsymbol{cx}^{\boldsymbol{2}}$ | 0.96 | 0.153 |
|  | Gaussion | $\boldsymbol{y=a+bcos(cx+d)}$ | 0.96 | 0.163 |
|  | Quadratic Fit | $\boldsymbol{y=a}\boldsymbol{e}^{\boldsymbol{-(x-b)}^{\boldsymbol{2}}\mathbf{/2}\boldsymbol{c}^{\boldsymbol{2}}}$ | 0.95 | 0.166 |
| 40% | MMF | $\boldsymbol{y=}\frac{\boldsymbol{a+bx}}{\boldsymbol{1+cx+d}\boldsymbol{x}^{\boldsymbol{2}}}$ | 0.97 | 0.191 |
|  | Rational Function | $\boldsymbol{y=a-}\boldsymbol{be}^{\boldsymbol{-cx^d}}$ | 0.96 | 0.219 |
|  | Gaussion | $\boldsymbol{y=a+bx+}\boldsymbol{cx}^{\boldsymbol{2}}$ | 0.90 | 0.330 |
|  | Quadratic Fit | $\boldsymbol{y=a+bcos(cx+d)}$ | 0.90 | 0.349 |

**Table S2.** Comparison of correlation coefficients and error rate for brix changes

| Conc. | Correlation coefficient (R^2^) | Error  (S) | Correlation coefficient (R^2^) | Error  (S) | Correlation coefficient (R^2^) | Error  (S) | Correlation coefficient (R^2^) | Error  (S) |
| --- | --- | --- | --- | --- | --- | --- | --- | --- |
| 20% | MMF Model | | Rational Function | | Gaussion Model | | Quadratic Fit | |
|  | 0.98^b^ | 0.12^a^ | 0.86^a^ | 0.39^b^ | 0.86^a^ | 0.38^b^ | 0.87^a^ | 0.38^b^ |
| 30% | Rational Function | | Quadratic Fit | | Sinusiodal Fit | | Gaussion Model | |
|  | 0.98^a^ | 0.10^a^ | 0.96^a^ | 0.15^a^ | 0.96^a^ | 0.16^a^ | 0.95^a^ | 0.16^a^ |
| 40% | Rational Function | | Weibull Model | | Quadratic Fit | | Sinusiodal Fit | |
|  | 0.97^a^ | 0.19^a^ | 0.96^a^ | 0.22^a^ | 0.90^a^ | 0.33^b^ | 0.90^a^ | 0.35^b^ |

**Table S3.** Presented models for pH changes with the growth of *A. pasteurianus* in alcoholic extracts

| **Extract** | **Model** | **Equation** | **Correlation coefficient (R^2^)** | **Error )S(** |
| --- | --- | --- | --- | --- |
| 20% | Heat Capacity | $\boldsymbol{y}\boldsymbol{=a+bx+}\frac{\boldsymbol{c}}{\boldsymbol{x}^{\boldsymbol{2}}}$ | 0.93 | 0.263 |
|  | Gaussion | $\boldsymbol{y=a}\boldsymbol{e}^{\boldsymbol{-(x-b)}^{\boldsymbol{2}}\mathbf{/2}\boldsymbol{c}^{\boldsymbol{2}}}$ | 0.93 | 0.265 |
|  | Quadratic Fit | $\boldsymbol{y=a+bx+}\boldsymbol{cx}^{\boldsymbol{2}}$ | 0.93 | 0.266 |
|  | Rational Function | $\boldsymbol{y=}\frac{\boldsymbol{a+bx}}{\boldsymbol{1+cx+d}\boldsymbol{x}^{\boldsymbol{2}}}$ | 0.93 | 0.277 |
| 30% | Rational Function | $\boldsymbol{y=}\frac{\boldsymbol{a+bx}}{\boldsymbol{1+cx+d}\boldsymbol{x}^{\boldsymbol{2}}}$ | 0.95 | 0.194 |
|  | Quadratic Fit | $\boldsymbol{y=a+bx+}\boldsymbol{cx}^{\boldsymbol{2}}$ | 0.81 | 0.345 |
|  | Heat Capacity | $\boldsymbol{y=a+bx+}\frac{\boldsymbol{c}}{\boldsymbol{x}^{\boldsymbol{2}}}$ | 0.81 | 0.346 |
|  | Gaussion | $\boldsymbol{y=a}\boldsymbol{e}^{\boldsymbol{-(x-b)}^{\boldsymbol{2}}\mathbf{/2}\boldsymbol{c}^{\boldsymbol{2}}}$ | 0.81 | 0.366 |
| 40% | Rational Function | $\boldsymbol{y=}\frac{\boldsymbol{a+bx}}{\boldsymbol{1+cx+d}\boldsymbol{x}^{\boldsymbol{2}}}$ | 0.95 | 0.254 |
|  | Weibull Model | $\boldsymbol{y=a-}\boldsymbol{be}^{\boldsymbol{-cx^d}}$ | 0.80 | 0.455 |
|  | Quadratic Fit | $\boldsymbol{y=a+bx+}\boldsymbol{cx}^{\boldsymbol{2}}$ | 0.80 | 0.456 |
|  | Sinusiodal Fit | $\boldsymbol{y=a+bcos(cx+d)}$ | 0.80 | 0.457 |

**Table S4.** Comparison of correlation coefficients and error rate for pH changes

| Conc. | Correlation coefficient (R^2^) | Error  (S) | Correlation coefficient (R^2^) | Error  (S) | Correlation coefficient (R^2^) | Error  (S) | Correlation coefficient (R^2^) | Error  (S) |
| --- | --- | --- | --- | --- | --- | --- | --- | --- |
| 20% | Heat Capacity Model | | Gaussion Model | | Quadratic Fit | | Rational Function | |
|  | 0.93^a^ | 0.26^a^ | 0.93^a^ | 0.26^a^ | 0.93^a^ | 0.26^a^ | 0.93^a^ | 0.27^a^ |
| 30% | Rational Function | | Quadratic Fit | | Heat Capacity Model | | Gaussion Model | |
|  | 0.95^b^ | 0.15^a^ | 0.81^a^ | 0.34^b^ | 0.81^a^ | 0.34^b^ | 0.81^a^ | 0.36^b^ |
| 40% | Rational Function | | Weibull Model | | Quadratic Fit | | Sinusiodal Fit | |
|  | 0.95^b^ | 0.25^a^ | 0.80^a^ | 0.45^b^ | 0.80^a^ | 0.45^b^ | 0.80^a^ | 0.46^b^ |

**Table S5.** Presented models for changes in acidity with the growth of *A. pasteurianus* in alcoholic extracts

| **Extract** | **Model** | **Equation** | **Correlation coefficient (R^2^)** | **Error )S(** |
| --- | --- | --- | --- | --- |
| 20% | Richards | $\boldsymbol{y=}\frac{\boldsymbol{a}}{\left( \boldsymbol{1+}\boldsymbol{e}^{\boldsymbol{b-cx}} \right)^{\boldsymbol{1/d}}}$ | 0.99 | 0.263 |
|  | Weibull | $\boldsymbol{y=a-}\boldsymbol{be}^{\boldsymbol{-c}\boldsymbol{x}^{\boldsymbol{d}}}$ | 0.98 | 0.265 |
|  | MMF | $\boldsymbol{y=}\frac{\boldsymbol{ab+}\boldsymbol{cx}^{\boldsymbol{d}}}{\boldsymbol{b+}\boldsymbol{x}^{\boldsymbol{d}}}$ | 0.98 | 0.266 |
|  | Logistic | $\boldsymbol{y=}\frac{\boldsymbol{a}}{\boldsymbol{1+b}\boldsymbol{e}^{\boldsymbol{-cx}}}$ | 0.98 | 0.277 |
| 30% | Gompertz | $\boldsymbol{y=a}\boldsymbol{e}^{\boldsymbol{-}\boldsymbol{e}^{\boldsymbol{b-cx}}}$ | 0.95 | 0.194 |
|  | Logistic | $\boldsymbol{y=}\frac{\boldsymbol{a}}{\boldsymbol{1+b}\boldsymbol{e}^{\boldsymbol{-cx}}}$ | 0.96 | 0.345 |
|  | Weibull | $\boldsymbol{y=a-}\boldsymbol{be}^{\boldsymbol{-cx^d}}$ | 0.97 | 0.346 |
|  | MMF | $\boldsymbol{y=}\frac{\boldsymbol{ab+}\boldsymbol{cx}^{\boldsymbol{d}}}{\boldsymbol{b+}\boldsymbol{x}^{\boldsymbol{d}}}$ | 0.95 | 0.366 |
| 40% | Gompertz | $\boldsymbol{y=a}\boldsymbol{e}^{\mathbf{-}\boldsymbol{e}^{\boldsymbol{b-cx}}}$ | 0.96 | 0.254 |
|  | Logistic | $\boldsymbol{y=}\frac{\boldsymbol{a}}{\boldsymbol{1+b}\boldsymbol{e}^{\boldsymbol{-cx}}}$ | 0.95 | 0.455 |
|  | Weibull | $\boldsymbol{y=a-}\boldsymbol{be}^{\boldsymbol{-cx^d}}$ | 0.95 | 0.456 |
|  | Richards | $\boldsymbol{y=}\frac{\boldsymbol{a}}{\left( \boldsymbol{1+}\boldsymbol{e}^{\boldsymbol{b-cx}} \right)^{\boldsymbol{1/d}}}$ | 0.95 | 0.456 |

**Table S6.** Comparison of correlation coefficients and error rate for acidity changes

| Conc. | Correlation coefficient (R^2^) | Error  (S) | Correlation coefficient (R^2^) | Error  (S) | Correlation coefficient (R^2^) | Error  (S) | Correlation coefficient (R^2^) | Error  (S) |
| --- | --- | --- | --- | --- | --- | --- | --- | --- |
| 20% | Richards Model | | Weibull Model | | MMF Model | | Logistic Model | |
|  | 0.99^a^ | 0.10^a^ | 0.98^a^ | 0.12^a^ | 0.98^a^ | 0.13^a^ | 0.98^a^ | 0.14^a^ |
| 30% | Gompertz Relation | | Logistic Model | | Weibull Model | | MMF Model | |
|  | 0.95^a^ | 0.18^a^ | 0.96^a^ | 0.18^a^ | 0.97^a^ | 0.19^a^ | 0.95^a^ | 0.20^a^ |
| 40% | Gompertz Relation | | Logistic Model | | Weibull Model | | Richards Model | |
|  | 0.96^a^ | 0.21^a^ | 0.95^a^ | 0.21^a^ | 0.95^a^ | 0.22^a^ | 0.95^a^ | 0.22^a^ |

**Table S7.** Presented models for alcohol changes with the growth of *A. pasteurianus* in alcoholic extracts

| **Extract** | **Model** | **Equation** | **Correlation coefficient (R^2^)** | **Error )S(** |
| --- | --- | --- | --- | --- |
| 20% | MMF | $\boldsymbol{y=}\frac{\boldsymbol{ab+}\boldsymbol{cx}^{\boldsymbol{d}}}{\boldsymbol{b+}\boldsymbol{x}^{\boldsymbol{d}}}$ | 0.97 | 0.165 |
|  | Gaussion | $\boldsymbol{y=a}\boldsymbol{e}^{\boldsymbol{-(x-b)}^{\boldsymbol{2}}\boldsymbol{/}{\boldsymbol{2}\boldsymbol{c}}^{\boldsymbol{2}}}$ | 0.97 | 0.170 |
|  | Rational Function | $\boldsymbol{y=}\frac{\boldsymbol{a+bx}}{\boldsymbol{1+cx+d}\boldsymbol{x}^{\boldsymbol{2}}}$ | 0.97 | 0.170 |
|  | Sinusiodal Fit | $\boldsymbol{y=a+bcos(cx+d)}$ | 0.97 | 0.179 |
| 30% | Rational Function | $\boldsymbol{y=}\frac{\boldsymbol{a+bx}}{\boldsymbol{1+cx+d}\boldsymbol{x}^{\boldsymbol{2}}}$ | 0.98 | 0.105 |
|  | Quadratic Fit | $\boldsymbol{y=a+bx+}\boldsymbol{cx}^{\boldsymbol{2}}$ | 0.83 | 0.341 |
|  | Gaussion | $\boldsymbol{y=a}\boldsymbol{e}^{\boldsymbol{-(x-b)}^{\boldsymbol{2}}\mathbf{/2}\boldsymbol{c}^{\boldsymbol{2}}}$ | 0.81 | 0.355 |
|  | Sinusiodal Fit | $\boldsymbol{y=a+bcos(cx+d)}$ | 0.83 | 0.360 |
| 40% | Rational Function | $\boldsymbol{y=}\frac{\boldsymbol{a+bx}}{\boldsymbol{1+cx+d}\boldsymbol{x}^{\boldsymbol{2}}}$ | 0.98 | 0.136 |
|  | Quadratic Fit | $\boldsymbol{y=a+bx+}\boldsymbol{cx}^{\boldsymbol{2}}$ | 0.83 | 0.423 |
|  | Sinusiodal Fit | $\boldsymbol{y=a+bcos(cx+d)}$ | 0.84 | 0.448 |
|  | Gaussion | $\boldsymbol{y=a}\boldsymbol{e}^{\boldsymbol{-(x-b)}^{\boldsymbol{2}}\mathbf{/2}\boldsymbol{c}^{\boldsymbol{2}}}$ | 0.81 | 0.445 |

**Table S8.** Comparison of correlation coefficients and error rate for alcohol changes

| Conc. | Correlation coefficient (R^2^) | Error  (S) | Correlation coefficient (R^2^) | Error  (S) | Correlation coefficient (R^2^) | Error  (S) | Correlation coefficient (R^2^) | Error  (S) |
| --- | --- | --- | --- | --- | --- | --- | --- | --- |
| 20% | MMF Model | | Gaussion Model | | Rational Function | | Sinusiodal Fit | |
|  | 0.97^a^ | 0.16^a^ | 0.97^a^ | 0.17^a^ | 0.97^a^ | 0.17^a^ | 0.97^a^ | 0.18^a^ |
| 30% | Rational Function | | Quadratic Fit | | Gaussion Model | | Sinusiodal Fit | |
|  | 0.98^b^ | 0.10^a^ | 0.83^a^ | 0.34^b^ | 0.81^a^ | 0.35^b^ | 0.83^a^ | 0.36^b^ |
| 40% | Rational Function | | Quadratic Fit | | Sinusiodal Fit | | Gaussion Model | |
|  | 0.98^b^ | 0.13^a^ | 0.83^a^ | 0.42^b^ | 0.83^a^ | 0.44^b^ | 0.81^a^ | 0.44^b^ |
